# The Nursing of the Future: combining Digital Health and the Leadership of Nurses

**DOI:** 10.1590/1518-8345.0000.3338

**Published:** 2020-06-19

**Authors:** Luís Velez Lapão

**Affiliations:** 1 Global Health and Tropical Medicine (GHTM), WHO Collaborating Center for Health Workforce Policy and Planning, Instituto de Higiene e Medicina Tropical, Lisboa, Portugal.; 2 President of the Conselho Geral da Escola Superior de Enfermagem de Lisboa, Lisboa, Portugal.

**Figure:**
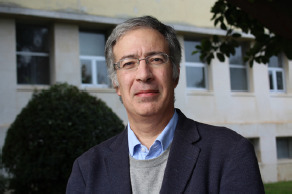


The practice of caring for sick people has existed since ancient times. Contemporarily, we owe to Florence Nightingale and other exponents the conception of Nursing as a profession, being fundamental in guaranteeing health care, which is increasingly developed by multidisciplinary teams with the potential for greater proximity to people through the use of digital technologies, especially in an increasingly global world^(^ 1 ^)^.

The insufficient number of nurses in the world is an identified problem and has been a global concern. However, there is a lack of leaders assuming the necessary changes in sanitary practices, management, teaching and health policies in general and, above all, in promoting multidisciplinary teamwork. In this sense, the focus is now on the leadership of a new generation of nurses, who can reinforce the aspects of ethics and values as the central axis of care with the interactivity of its users in the health systems and, for this, is fundamental to enhance the leadership of experienced nurses. The World Health Organization and the *Global Advisory Panel on the Future of Nursing* suggest that effective communication between health professionals and users of health services is imperative, as well as good technical performance, with integrity and humanism, aligned with the Sustainable Development objectives^(^ 2 ^)^. A set of structural and organizational barriers limit nurses in responding to rapid changes in health. In 2008, The Robert Wood Johnson Foundation and the Institute of Medicine organized an initiative to visualize the future of the nursing profession, establishing four recommendations:

- Nurses should promote care to the fullest extent of their specialty, in the different contexts of health services.
- Nurses must obtain high levels of knowledge through an improved educational system that promotes continuous progression, to improve the quality of care, with the potential to make the nursing workforce more diverse, particularly in terms of gender and race/ethnicity.
- Interprofessional practice should be the primary focus in the reformulation of health systems that do not adopt universal systems.
- The nurse must participate in the planning of the workforce and in the development of policies for an improved information system.

Nursing is the profession that has the largest contingent of workforce in different health systems worldwide. In this sense, the nurse’s leadership must play a fundamental role in the organization of work and in the development of innovative solutions. The guiding principles of responsibility, the respect for specialization and the commitment to the objectives of quality and proximity to users of health services will allow the nursing leadership to contribute to the implementation of innovative digitally based solutions in nursing^(^ 3 ^)^.

In this way, the maturity of digital health transformation is an opportunity to qualify nursing work. However, it is also a challenge, as it requires the nursing workforce to develop specific skills in the digital area. The Internet (e.g. Applications) allows unique conditions to strengthen the bond with users of health services. The creation of digital health services such as monitoring users from a distance will allow a better response to health care, eventually leading to the creation of “precision nursing”. Thus, the permanent and continuous qualification of nursing professionals must be involved with digital technologies in order to establish a skilled workforce to resolutely meet the future demands of an increasingly computerized world^(^ 4 ^)^.

The Nursing of the future will be supported by increasingly qualified professionals, focused on advanced practice, whose knowledge will support their leadership in the reorganization of the practice of care, in partnership with other professionals and with greater proximity to the users of health services. The adoption of therapeutic measures will be facilitated by the digital systems through “smart” clinical protocols, which are consensually interprofessional and will allow to highlight the teamwork with a more transparent way, in an effective link to the health service user. The new sensors (e.g Internet of Things, wearables), new technological networks, robots, 3-D printing and, above all, the most sophisticated and complex decision support systems for care and management practice, will support a more interactive in-depth research with health service users^(^ 1 ^)^. The interaction digital proficient nurses with users will put them at the center of care and make them more active as managers of shared self-care in health. The Digital Nursing Services will be able to reinforce the nurse’s capacity to interact with the users of the health services and their families in order to promote better adherence to therapies and support the “social capital” that dynamizes healthy communities. After gathering consistent data, artificial intelligence can be an ally of nurses in health care management, helping them to anticipate and identify situations that may constitute potential or real health problems for users of health services^(^ 5 ^)^.

The in-depth knowledge of health organizations will allow nurses to use the science of Design to promote innovation in health services^(^ 1 ^)^. Thus, the nurse, acting in an interprofessional manner, will promote the care process and the interaction with users of health services in a digital system that will constitute a new paradigm of nursing care.
